# Challenges and Considerations for Reducing Diabetes Distress and Fear of Hypoglycemia in Parents of Youth With Type 1 Diabetes During the COVID-19 Pandemic

**DOI:** 10.2196/25106

**Published:** 2021-04-23

**Authors:** Alexandra Monzon, Nicole Kahhan, Arwen Marker, Susana Patton

**Affiliations:** 1 Clinical Child Psychology Program University of Kansas Lawrence, KS United States; 2 Center for Healthcare Delivery Science Nemours Children’s Health System Jacksonville, FL United States

**Keywords:** type 1 diabetes, parents, children, diabetes distress, fear of hypoglycemia, COVID-19, telehealth, diabetes, challenge, youth, young adults

## Abstract

Type 1 diabetes management can be challenging for children and their families. To address psychosocial concerns for parents of youth with type 1 diabetes, we developed two parent-focused interventions to reduce their diabetes distress and fear of hypoglycemia. Our team conducted several of these interventions during the early stages of the COVID-19 pandemic and recognized a need to make timely adjustments to our interventions. In this viewpoint article, we describe our experience conducting these manualized treatment groups during the pandemic, the range of challenges and concerns specific to COVID-19 that parents expressed, and how we adjusted our approach to better address parents’ treatment needs.

## Introduction

The daily self-management of type 1 diabetes (T1D) is complex and unrelenting. It involves regular glucose monitoring, healthy eating and carbohydrate assessment, insulin administration via syringe or continuous subcutaneous insulin infusion, and physical activity [1]. The goal of modern T1D self-management is to maintain near-normal glucose levels [1]. However, for many families of youth with T1D, this goal can be very hard to achieve [2,3]. There is evidence that many parents of youth with T1D experience diabetes distress and fear of hypoglycemia, which may negatively impact their functioning and quality of life [4-6]. Further, parents who report maladaptive coping strategies also report decreases in mental and physical health [7]. In our own work, we found that nearly 60% of parents of young children with T1D (<6 years) report at least a moderate level of hypoglycemia fear (FH) [8,9]. In addition, our data and the results of published studies suggest that between 10%-74% of parents report diabetes distress (DD) [5,6,10,11]. It is because of these relatively high prevalence rates for FH and DD among parents that we developed two novel parent-focused interventions to increase adaptive coping and reduce their FH and DD.

## Reducing Emotional Distress to Childhood Hypoglycemia in Parents

Reducing Emotional Distress to Childhood Hypoglycemia in Parents (REDCHiP) is a manualized and closed group video-based telehealth intervention [12]. REDCHiP includes 10 sessions (7 group sessions and 3 individual sessions) delivered over approximately 13 weeks. During REDCHiP, parents do the following: (1) review T1D education and problem-solving to increase self-efficacy for the management of hypoglycemic events, (2) learn age-appropriate behavioral parenting strategies to manage child behaviors in the context of T1D care, and (3) learn cognitive-behavioral therapy (CBT) strategies to enhance coping with fear and stress related to hypoglycemia [12]. In our pilot work, parents receiving the REDCHiP intervention showed significant reductions in their report of FH (*P*=.003, *d*=1.01) and parenting stress (*P*=.003, *d*=0.85), and children with glycated hemoglobin (HbA_1c_) levels >7.5% prior to REDCHiP showed a significant reduction in their HbA_1c_ levels (*P*=.049, *d*=0.43) after participating in the intervention [13]. Based on these promising results, we are now in the process of conducting a larger randomized clinical trial to test the efficacy of our REDCHiP intervention versus a relevant attention control group [14].

## Cognitive Adaptations to Reduce Emotional Stress

To address parents’ perceptions of DD, we developed Cognitive Adaptations to Reduce Emotional Stress (CARES) based on the theory of stress and coping [15-17]. Like REDCHiP, CARES is a manualized video-based telehealth intervention that includes weekly closed group sessions delivered over 8 or 12 weeks, depending on distress severity. In CARES, we use principles of CBT to teach parents how to identify unhelpful thoughts, feelings, and behaviors specific to T1D and how to use both mindfulness-based strategies (eg, meditation, being in the moment) and behavioral activation to manage their negative thoughts and feelings related to T1D. Our preliminary data suggest a significant reduction in parents’ report of DD as a result of CARES (*d*=0.71) [18] and we are currently in the process of applying for additional grant funding to conduct a larger randomized clinical trial of this intervention.

## Intervention Impacts of COVID-19

In early 2020, the United States, like many other countries, faced an unprecedented public health event with the rapid spread of COVID-19. For some families of youth with T1D, COVID-19 may be a new stressor that disrupts routine diabetes care and negatively impacts family engagement with optimal T1D self-management behaviors, including healthy eating, physical activity, and adequate insulin administration. In addition, exposure to this stressor could increase the risk of youth and/or their parents developing symptoms of anxiety and depression or exacerbate symptoms already present. Previous studies suggest that parent stress and internalizing symptoms may increase their child’s risk for developing similar symptoms unless the family engages in more adaptive coping methods [19]. Further, families may also face increased fear of exposure to COVID-19, making previously typical activities of daily life (eg, shopping, work/school, recreation/physical activity) more difficult to accomplish or seemingly riskier to do. Per the Centers for Disease Control and Prevention (CDC), diabetes is a risk factor for severe illness [20], and emerging data from the T1D Exchange suggest persons with T1D who contract COVID-19 may be vulnerable to experiencing acute T1D-specific events including severe hyperglycemia and diabetes ketoacidosis (DKA) [21]. Thus, it is possible that some parents of children with T1D may be experiencing added fear and/or distress because of COVID-19 beyond that of the general population. During the early spread of COVID-19 in the United States, our team recognized a need to make some timely adjustments to our REDCHiP and CARES interventions to help parents reduce their FH and DD in the context of COVID-19. In this viewpoint article, we describe our experience conducting these manualized treatment groups with parents of youth with T1D during the pandemic, the range of challenges and concerns specific to COVID-19 that parents brought up in groups, and how we, in turn, adjusted our approach to better address parents’ experiences and treatment needs.

## Participants and Author Viewpoint

All parents who participated in the treatment groups had a child with a confirmed diagnosis of T1D for at least 6 months who was following an intensive insulin regimen. We recruited families of youth between the ages of 1-6 years to the CARES intervention across sites in the Midwest region of the United States. We also recruited families of youth between the ages of 5-12 years to the REDCHiP intervention across sites in the Midwest and Southeast regions of the United States. Each treatment group contained 3-4 members. As part of the established procedures for both trials, we video recorded the telehealth sessions to allow for coding of treatment integrity. However, these recordings also enabled us to reflect on the parents’ view and observe the adjustments that group leaders made in the groups they led. The challenges and adaptations discussed in this viewpoint were not objectively measured nor part of a formal qualitative study. Rather, this viewpoint article is based on experiences of 4 treatment groups (1 CARES and 3 REDCHiP) and our consensus regarding the specific concerns parents raised in the CARES and REDCHiP treatment groups during the early months of the COVID-19 pandemic in the United States and how we observed group leaders adapt the intervention content during the onset of the pandemic to better address parents’ concerns.

## COVID-19–Related Challenges and Concerns

The participants in our active treatment groups reported several concerns and challenges when caring for their child with T1D during the onset of the pandemic. Not surprisingly, a main concern raised by parents was the perceived risk their child with T1D may contract COVID-19, which could increase their risk of negative health outcomes. In the early months of the COVID-19 pandemic, parents reported increased stress and anxiety regarding the safety of their child (eg, one parent even remarked, “[I] see germs everywhere”). Parents commented that their child with T1D was in a high-risk group, a notion also frequently highlighted by the media. Moreover, because parents had learned of a possible association between suboptimal diabetes management and COVID-19, they felt an increased pressure to maintain tighter glycemic control for their child. Some parents also expressed significant concern that their child’s T1D would be difficult to manage if either the parent or child became sick. Indeed, families specifically noted heightened anxiety about the challenges of managing out-of-range blood glucose values when their young child with T1D was sick in the past and this seemed to exacerbate their fears about possible COVID-19 illness. One family in particular, who had previously struggled to manage diabetes when their child was sick, reported significantly changing their lifestyle during cold and flu season in other years to reduce perceived risk (eg, avoiding sport activities, libraries). Parents reported that the stress associated with their child becoming sick further intensified as they started to seek out more information about the transmission of COVID-19 (eg, airborne versus surface contact) and when trying to maintain awareness of current recommendations (ie, when/where to use a face covering) during a time when new and sometimes conflicting information was continuously available.

In addition to anxiety about COVID-19 risk, many families faced a significant challenge when stay-at-home orders took effect and schools and local businesses began to shut down, impacting their typical routines. Maintaining a consistent routine can be an important component of optimal diabetes management [1]; it can also be helpful when raising a young child [22,23]. Therefore, adjusting to a substantial change in routine was challenging for some parents who previously relied on school schedules for beneficial structure in managing their child’s daily T1D regimen. Some caregivers reported increased stress due to taking on increased childcare and diabetes tasks during the day. Parents also lost access to other childcare options (ie, daycare, nannies, or extended family/other caregivers), which may have increased disease management burden as they juggled diabetes treatment tasks, online teaching, childcare, and their own work-related responsibilities. Further, many parents noted fewer opportunities for their child to engage in safe and structured physical activity and indicated that they were concerned this would negatively impact their child’s glucose levels. Parents also noted their own difficulty engaging in behavioral activation strategies (ie, regular and enjoyable activities to increase mood) or healthy lifestyle behaviors as a result of stay-at-home orders and reduced access to activities they would typically choose to do. Even after stay-at-home restrictions ended for some families, parents noted a period of suboptimal glycemic control when they returned to the office after working remotely for several months. These parents expressed frustration that changing schedules negatively impacted diabetes management and indicated heightened worry and guilt about returning to the office and the potential risk of contracting or exposing their child to COVID-19.

Another major challenge of COVID-19 discussed during the treatment groups was each family’s experience of social isolation. Several parents reported they felt isolated from friends and unable to use their typical resources to manage daily stress (ie, gym, church, social gatherings, self-care outside of home). Similarly, several parents reported they restricted their child’s play with peers, contributing to their child’s increased sense of isolation. Parents expressed new worries when they considered allowing their child to interact with other people. Moreover, they reported feeling guilty when they did not allow their child to play with a peer or visit extended family during the stay-at-home orders. Feelings of isolation were not only specific to social activities but also included managing T1D. One parent in particular felt isolated during the stay-at-home order because her partner did not assist with T1D care and she had come to rely on her child’s school nurse for help with diabetes management during the school day. Unfortunately, during the stay-at-home orders she was unable to access assistance from the school nurse. Some parents also reported that it was challenging to attend the treatment group sessions during the stay-at-home orders and that they felt overwhelmed by all their responsibilities. In fact, several families who had previously expressed interest in participating in a group declined to participate during the stay-at-home orders, citing difficulty in attending the treatment groups while simultaneously having all family members at home. Further, we had parents frequently reschedule their meeting times to accommodate changes in their daily schedules. Lastly, another untimely challenge that parents reported was COVID-19–related job loss or furloughs, which in some cases had a downstream impact on the family’s financial stability and insurance status. However, even parents who did not experience job loss reported concerns about their job security or their ability to find a new job and how that could impact their family’s insurance status and ability to pay for T1D management supplies.

## Positive Outcomes and Family Resilience

Despite the negative impacts of COVID-19 on many families, group leaders also noted positive outcomes and family resilience during this unprecedented time. Some families did not express specific concerns for their child related to COVID-19 and adapted to changes in lifestyle and schedules smoothly. Some parents even noted an improvement in their child’s blood glucose levels, which they attributed to their increased monitoring of T1D management tasks during stay-at-home orders. Families expressed gratitude for the support they received from the group members and group leaders. Even in the context of COVID-19–related challenges and concerns, many families continued to arrive to each session and remained engaged in group discussions. Several parents reported that their group participation increased as their schedules became more flexible as a result of working from home. Lastly, parents expressed appreciation for the extra family time they experienced related to stay-at-home orders.

## Treatment Adjustments and Considerations

To address the unique challenges and concerns raised during the treatment groups, and to continue to reduce parent fear and distress, group leaders made small adjustments in their approach, using clinical judgment. One common adjustment was to incorporate strategies consistent with Acceptance and Commitment Therapy [24]. In the context of COVID-19, these strategies seemed particularly appropriate, especially given the uncertainty and the changes happening outside of participants’ control. For example, when parents talked about their child feeling isolated from peers and unhappy, group leaders individually determined that problem-solving and information seeking might not provide parents the desired relief from negative feelings. Instead, the group leaders tried acceptance and commitment strategies aimed at helping the parents to accept that their child could feel isolation from time to time during the stay-at-home order and to commit to moving forward in life based on their values. Similarly, specific to T1D care, when parents reported difficulty in managing their child’s diabetes, the group leaders aimed to increase parents’ acceptance and tolerance of temporary child blood glucose fluctuations during periods of transition, while still helping parents commit to actions aligned with an eventual return to more stable T1D management. Group leaders also employed these strategies to help parents process any feelings of guilt related to returning to the office or when discussing a family’s decision to reduce their level of isolation (eg, playing with neighborhood peers, cousins). Group leaders discussed pros and cons of accepting different imperfect outcomes, such as increased feelings of isolation or increased risk of exposure, and helped families consider how they could commit to the course that best fit their perceived needs (eg, reduced risk of infection, children’s social development).

In addition to adjusting some therapeutic strategies, group leaders commonly spent more time and emphasis on problem-solving than initially planned, especially when aiming to increase parents’ use of behavioral activation strategies and helping them to identify available activities that were considered safe during COVID-19. For example, group leaders reported spending a lot of time on problem-solving strategies to help parents socialize and spend time with friends or extended family in a manner that felt comfortable and was within the scope of public health recommendations (eg, outdoor socially distanced walks with a friend/neighbor). The group leaders encouraged parents to embrace creative ways to achieve personal self-care (eg, spa night at home) and to integrate positive coping techniques despite the unique challenges of COVID-19 (eg, weekly video conversations with friends/family, virtual church service, outdoor and socially distant activities). Lastly, a novel behavior that many parents engaged in during groups was to seek advice from the group leader or other parents on whether their child should return to school. In these situations, the group leaders helped parents use risk-assessment strategies that were not initially part of either manualized treatment. Fortunately, problem-solving and soliciting parent examples to work through during the group sessions were already typical activities for both the REDCHiP and CARES interventions, which helped the group leaders make these adjustments more seamlessly.

## Future Directions

After addressing unique COVID-19–related challenges within each treatment group, our team hypothesized that there could be an increased risk for some parents to remain inappropriately hypervigilant about their child’s health after COVID-19 subsides, and that this could be an important area of ongoing concern for families. Interestingly, group leaders noted that some parents with higher levels of pre-existing anxiety reported a decrease in anxiety related to the stay-at-home orders. In many cases, these parents reported that they thought they could easily meet their child’s needs without interacting with others and that having their child home would be more conducive to monitoring their child’s health and T1D management nearly continuously. Although this may seem like a short-term improvement in anxiety (and potentially T1D management), this avoidant-based coping strategy could lead to longer term risks and challenges, especially if families rely too heavily on this strategy and continue to resist leaving their home for work, school, or social/recreation activities. In the time of COVID-19, it might not have been recommended that these families expose themselves to situations that increased their anxiety (eg, going for a socially distanced walk with friends), but nonetheless, group leaders continued to challenge families to expose themselves to situations and try new activities just outside their comfort zone (but still in line with CDC and medical team recommendations) in an effort to reduce the likelihood that families might adopt a lifestyle of avoiding anxiety-producing situations.

Lastly, our a priori decision to run treatment groups via a videoconferencing platform enabled group leaders to continue with scheduled sessions as stay-at-home orders took place, without a break in either treatment group. The use of telehealth services has recently become a large focus, in both medical and mental health service delivery, and this shift in service delivery may continue well into the future now that many families have experience with a telehealth platform. Although some services will return to in-person delivery as social distancing requirements are reduced, we would encourage providers to advocate that telehealth services remain an option for families. There are several benefits to continuing to provide telehealth services after COVID-19 subsides, such as increasing access to services for families living in rural areas, with limited transportation options, or with limited time available for such services. Although the available literature specific to the transition to telehealth services during the pandemic is limited, emerging research suggests telemedicine may be an effective approach for some families. For example, Garg and colleagues [25] presented a case example of using telemedicine to provide ongoing diabetes education to a pediatric patient with new-onset T1D. Their conclusion was that a telemedicine approach could be well-suited to families who use T1D devices (ie, insulin pump, continuous glucose monitor) where it is feasible to collect data remotely. Thus, the opportunities videoconferencing and telehealth affords us may continue to improve our ability to provide effective services to youth and families and reduce disparities in health care access both now and into the future.

## Conclusions

During the global COVID-19 pandemic, families of children with T1D faced new challenges, including widespread anxiety and activity restrictions to avoid COVID-19 exposure, while concurrently demonstrating marked resilience. Our research team was fortunate to work closely with families during this uncertain time through the REDCHiP and CARES group-based telehealth interventions. With some adjustments (ie, increased scheduling flexibility, greater focus on acceptance strategies, and additional time spent on problem-solving), we saw that parents continued to attend our treatment groups and to show individual success in managing negative affect related to T1D. As the COVID-19 pandemic evolves, we anticipate new concerns requiring further intervention or adjustment, such as difficulties returning to activities previously avoided to reduce COVID-19 risk, fluctuations in blood glucose following changes in routines, and/or increased burnout as many parents continue to shoulder responsibilities for childcare, school, T1D management, and their own work with no immediate end in sight. Further, formal qualitative studies are needed to intentionally assess the concerns we present in this viewpoint as the information provided was not the result of planned data collection. Future researchers and clinicians may consider formally assessing these concerns among patients and families to understand the extent to which these concerns impact daily functioning. We hope for the continued (and even more widespread) use of telehealth to deliver interventions to reduce anxiety and distress for families and children with T1D. Although research on the impacts of COVID-19 on families with children with T1D may be underway, it will also be important to exchange more anecdotal perspectives during this period of rapid change.

## Abbreviations

CARES: Cognitive Adaptations to Reduce Emotional Stress
CBT: cognitive-behavioral therapy
CDC: Centers for Disease Control and Prevention
DD: diabetes distress
DKA: diabetes ketoacidosis
FH: hypoglycemia fear
HbA_1c_: glycated hemoglobin
REDCHiP: Reducing Emotional Distress to Childhood Hypoglycemia in Parents
T1D: type 1 diabetes
